# OrthoDB v11: annotation of orthologs in the widest sampling of organismal diversity

**DOI:** 10.1093/nar/gkac998

**Published:** 2022-11-09

**Authors:** Dmitry Kuznetsov, Fredrik Tegenfeldt, Mosè Manni, Mathieu Seppey, Matthew Berkeley, Evgenia V Kriventseva, Evgeny M Zdobnov

**Affiliations:** Department of Genetic Medicine and Development, University of Geneva Medical School, Swiss Institute of Bioinformatics, rue Michel-Servet 1, 1211 Geneva, Switzerland; Department of Genetic Medicine and Development, University of Geneva Medical School, Swiss Institute of Bioinformatics, rue Michel-Servet 1, 1211 Geneva, Switzerland; Department of Genetic Medicine and Development, University of Geneva Medical School, Swiss Institute of Bioinformatics, rue Michel-Servet 1, 1211 Geneva, Switzerland; Department of Genetic Medicine and Development, University of Geneva Medical School, Swiss Institute of Bioinformatics, rue Michel-Servet 1, 1211 Geneva, Switzerland; Department of Genetic Medicine and Development, University of Geneva Medical School, Swiss Institute of Bioinformatics, rue Michel-Servet 1, 1211 Geneva, Switzerland; Department of Genetic Medicine and Development, University of Geneva Medical School, Swiss Institute of Bioinformatics, rue Michel-Servet 1, 1211 Geneva, Switzerland; Department of Genetic Medicine and Development, University of Geneva Medical School, Swiss Institute of Bioinformatics, rue Michel-Servet 1, 1211 Geneva, Switzerland

## Abstract

OrthoDB provides evolutionary and functional annotations of genes in a diverse sampling of eukaryotes, prokaryotes, and viruses. Genomics continues to accelerate our exploration of gene diversity and orthology is the most precise way of bridging gene functional knowledge with the rapidly expanding universe of genomic sequences. OrthoDB samples the most diverse organisms with the best quality genomics data to provide the leading coverage of species diversity. This update of the underlying data to over 18 000 prokaryotes and almost 2000 eukaryotes with over 100 million genes propels the coverage to another level. This achievement also demonstrates the scalability of the underlying OrthoLoger software for delineation of orthologs, freely available from https://orthologer.ezlab.org. In addition to the *ab-initio* computations of gene orthology used for the OrthoDB release, the OrthoLoger software allows mapping of novel gene sets to precomputed orthologs and thereby links to their annotations. The LEMMI-style benchmarking of OrthoLoger ensures its state-of-the-art performance and is available from https://lemortho.ezlab.org. The OrthoDB web interface has been further developed to include a pairwise orthology view from any gene to any other sampled species. OrthoDB-computed evolutionary annotations as well as extensively collated functional annotations can be accessed via REST API or SPARQL/RDF, downloaded or browsed online from https://www.orthodb.org.

## INTRODUCTION

Genomics continues to uncover the vast space of genetic sequences, but deciphering encoded gene functions remains a challenging problem. Orthologs are genes that have arisen by speciation, i.e. current representations of an ancestral gene, and they tend to preserve ancestral functions (1,2). Computational assessment of gene evolutionary relationships are much more scalable than functional experimentations, enabling us to tentatively extrapolate from the painstakingly acquired gene functional knowledge. However, scaling up the process of delineating gene orthology to the rate of accumulation of genomics data is also challenging. There exist many computational methods with varying trade-offs in precision, sensitivity and scalability as well as databases providing precomputed orthology data. Table 1 summarizes the current coverage of top databases (3–7), supplementing the Quest for Orthologs effort cataloguing orthology resources (https://questfororthologs.org/orthology_databases) (8). OrthoDB is based on the OrthoLoger software (https://orthologer.ezlab.org) and the LEMOrtho benchmarking framework (https://lemortho.ezlab.org) puts it in the context of state-of-the-art software for orthology inference (9–11). Sampling the widest coverage of species diversity, OrthoDB is the leading resource of precomputed gene orthology and collated functional annotations. It empowers comparative evolutionary studies and enables the most specific inferences of tentative gene functions.

**Table 1.** Phylogenetic coverage of major gene orthology resources

	OrthoDB.v11	OrthoDB.v10	eggNOG.v5	KEGG-OC	OMA
Release date	2022-09-15	2018-11-5	2018-11-12	2019-02-28	2021-12-1
Eukaryota	1935	1271	477	456	622
*- Metazoa*	*812*	*448*	*n.a*.	*n.a*.	*255*
*— Vertebrata*	*465*	*243*	*n.a*.	*n.a*.	*143*
*— Arthropoda*	*294*	*170*	*n.a*.	*n.a*.	*64*
*- Viridiplantae*	*171*	*117*	*n.a*.	*n.a*.	*74*
*- Fungi*	*782*	*549*	*n.a*.	*n.a*.	*206*
Bacteria	17 551	5609	4445	4880	1719
Archaea	607	404	168	278	155
Viruses	7962	6488	2502	0	119

## COVERAGE

This OrthoDB update provides analysis and annotation of over 100 million genes, increasing the species coverage to over 18 000 prokaryotes and almost 2000 eukaryotes, a very significant advance over our earlier records (Table 1). This quantity of data approaches the limits of computational resources; however, the genomics space is growing much faster, with many additional genomes already available and with many more yet to come. As in previous releases we sampled available genomes to cover the most diverse organisms with the best quality genomics data and the greatest number of functional annotation records. This approach to sampling of the genomic space allows more accurate inference of orthology and more accurate mapping of additional species to OrthoDB data. Mapping to precomputed orthologous groups and their annotations requires substantially fewer computational resources than *ab-initio* orthology predictions, and thus it should be the preferred way to link newly sequenced genomes to the current OrthoDB annotations.

**Figure 1.**
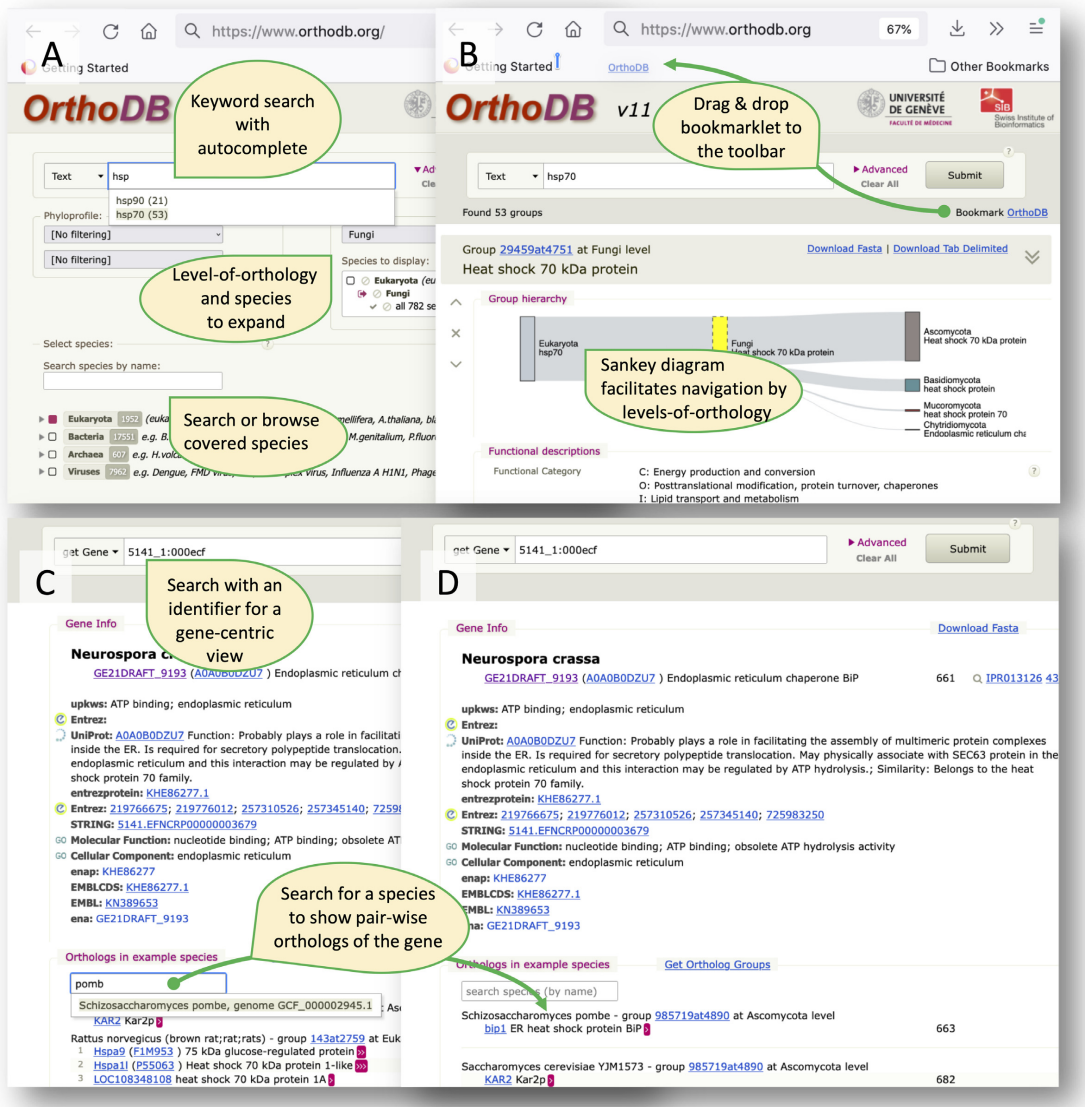
Elements of OrthoDB web interface: (**A**) the ‘Advanced’ section of the web interface enables user-tailored selection of organisms to focus on, specifying explicitly relevant levels of orthology, or phyloprofile filters. (**B**) the OG-centric results page shows an interactive Sankey diagram facilitating the navigation between the levels-of-orthology, and it presents a bookmarklet link that one can drag & drop to the browser toolbar for easy OrthoDB queries next time with the same filter settings. (**C**) the gene-centric view provides available gene annotations and a list of pair-wise orthologs in example species. (**D**) One can search for species of interest to list pair-wise orthologs in this species.

The details of selected organisms, including their assembly accession numbers, are available and searchable in the ‘Advanced’ section of the OrthoDB web user interface (Figure 1A). The orthology levels are defined according to the NCBI Taxonomy (12). Protein-coding gene translations are retrieved mostly from RefSeq and GenBank complete genomes (13,14). Our selection procedure identifies well-sampled taxonomic clusters having over 96% pairwise genomic identity using MASH (15) out of over 180 000 available complete genomes, and then we select the most annotated and BUSCO-complete (16) genome as a representative for each taxonomic cluster.

99% of the 100 million genes in this release contain non-trivial annotations, i.e. more than just its own sequence identifier. As the main source of the genomes for the release is RefSeq/GenBank, the protein sequence annotation available from NCBI is supplemented by mapped identifiers and textual descriptions from Uniprot (59%) (17), Ensembl/ENA (60%) (18), NCBI gid (34%), InterPro (49%) (19) and GO (42% total: 27% molecular function, 20% biological process, 20% cellular component) (20). These are further supplemented by mappings to EC categories (1.5%), KEGG genes (11%) and pathways (0.89%). The majority of human genes are mapped to NextProt (91%) (21), KEGG genes (91%) and pathways (36%) (22), as well as to disease-specific sources like OMIM (73%) (23). The total number of annotation sources is over 100, with a number of clade- or organism-specific sources, like FlyBase (24), VectorBase (25), ZFIN (26), MGI (27), SGD (28), etc.

As in previous releases, the abundance of gene annotations within each orthologous group (OG) is condensed into a one-line text description as previously described (29). This short description is often a clear message identifying the OG as a generalized representative of the gene at a given evolutionary level, thus helping users navigate the plethora of 11.6 Mio OGs available in this release. In addition to this semantic description, 49% of the OGs are also interlinked ‘horizontally’, i.e. with other functionally similar groups built at the same taxonomic level (aka ‘siblings’).

## ORTHOLOGER SOFTWARE

The central role of orthology for comparative studies of newly sequenced genomes and annotation of their genes creates a strong demand for a standalone software application. Delineation of orthologs requires first identifying homologs (genes sharing a common ancestry) and then grouping homologs originating from each of the genes of the last common ancestor of the species under consideration. Such genes, presumably having evolved from a single gene at a particular species radiation, constitute an orthologous group (OG). A reference to a particular species radiation, referred to as level-of-orthology, implies hierarchical relation among OGs (30–32). Hence orthologs are more finely-resolved for more closely related species, splitting earlier duplicated genes into homologous but distinct groups of orthologs. Practically, delineation of orthologs usually: (i) employs pairwise aligners to identify homologs across genomes (e.g. BLAST (33), MMseqs2 (34), DIAMOND (35)), (ii) then estimates evolutionary distances among the homologs to explicitly or implicitly reconcile gene and species trees and (iii) outputs groups of genes presumably originating from a single gene of the last common ancestor of the species under consideration. In OrthoDB, we rely on the OrthoLoger software that is configured to use MMseqs2 (34) for homology searches, relies on best-reciprocal-hits between each pair of species for identification of candidate orthologs (as best-reciprocal-hit is a proxy for reconciliation of the gene tree and a pair of species), and clusters these candidates into OGs.

**Figure 2.**
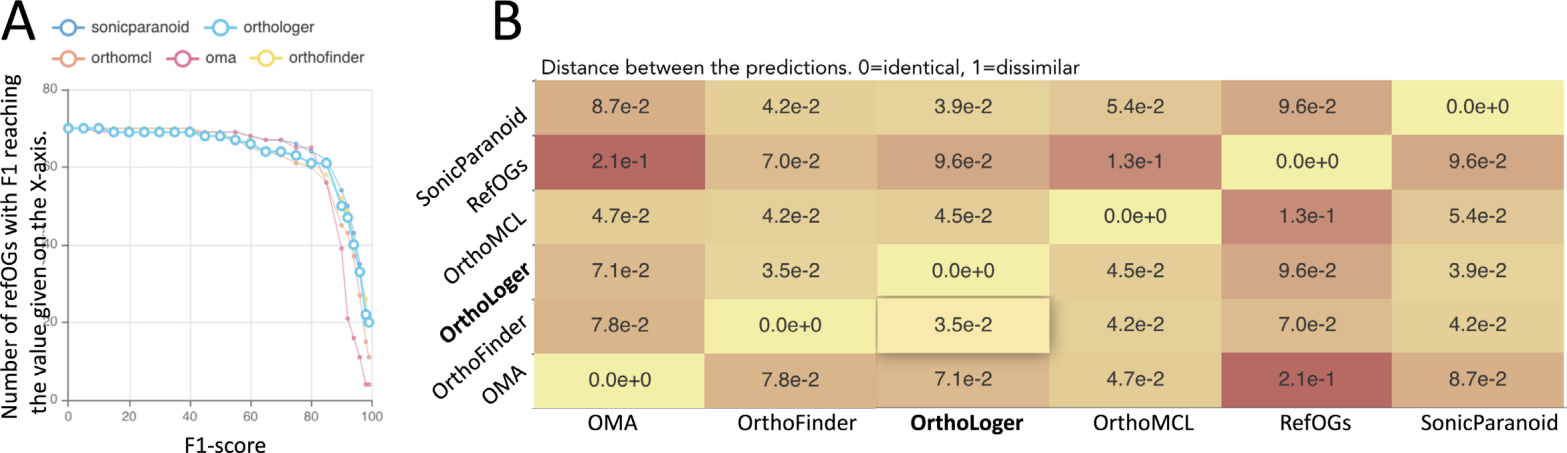
Benchmark (https://lemortho.ezlab.org/refogs) of the OrthoLoger software on refOGs. (**A**) The distribution of the F1 metric over refOGs shows that for the majority of refOGs F1 > 80%. (**B**) Concordance on ‘Variation of Information’ between the methods and RefOGs. The lower values indicate more similar classifications.

The complexity of the problem has prompted the development of many approaches. Each approach has limitations and software implementations have design choices that may affect results. This makes it necessary to benchmark the performance of complete procedures to allow users to select the most appropriate tool. The LEMMI benchmark framework (36) provides: (i) a continuous assessment, (ii) a dynamic presentation of results with supporting details and (iii) an effective distribution channel of tools through software containers. Extending this approach for Live Evaluation of Methods for Orthologs delineation (https://lemortho.ezlab.org) demonstrates the state-of-the-art performance of OrthoLoger (Figure 2). As the golden truth is not known for orthology, we used a set of expert-curated RefOGs (https://github.com/bio-mmanni/Open_Orthobench revised from (37,38)). There is also a very significant range of difficulty in orthology prediction for different gene families. This can be explained by the variance in acting selection pressure, affecting rates of gene duplication and losses as well as rates of sequence divergence. While single-copy orthologs are the easiest to identify, disambiguating relationships in large multi-gene families can be tricky, especially with frequent gene losses in addition to duplications. Thus, instead of reporting a single figure for precision, sensitivity, or the composite F1 score averaged over the sampled gene families as usually done (37), it is more illustrative to plot the value of the standard metrics on the x-axis with the counts of OGs where this metric is greater than x on the y-axis (Figure 2A). This generally recovers the comparative performance reported earlier (39) despite the revisions of refOGs and refinement of the methods. Considering the best OG combination overlap to refOGs, the distributions show very similar performance for OrthoLoger, OrthoFinder (9), and SonicParanoid (10) in terms of precision and sensitivity, with a minor bias of OrthoFinder towards higher sensitivity and of SonicParanoid towards higher specificity. The concordance between the tools beyond refOGs suggests that OrthoLoger results are closer to that of OrthoFinder than to the ones of SonicParanoid (Figure 2B).

OrthoLoger also implements a tree mode, using a user-provided species tree to hierarchically cluster OGs. This mode features better scalability with a very similar performance. However, we noted a substantial number of splits of refOGs (Table 2), i.e. when a refOG is reconstructed with more than one OG predicted by the methods, which is generally in agreement with our prior benchmarking (https://academic.oup.com/view-large/87032919, Table 2 in 39). Any automated predictions are susceptible to contain errors, and users should consider OrthoDB data as the first approximation to guide further investigations. Benchmarking in turn serves as quality control and it should reflect the degree of trust one should place in a chosen method. As the field is evolving we hope users will appreciate the more interactive approach to benchmarking now presented by LEMOrtho (https://lemortho.ezlab.org/refogs).

**Table 2.** Benchmark (https://lemortho.ezlab.org) of popular orthology methods versus revised RefOGs (37,38)

					RefOGs		
Method	Num. of OGs (RefOGs = 70)	RefOGs with **F1** ≥ 85%	RefOGs with precision ≥ 85%	RefOGs with recall ≥ 85%	Exact	Akin	Split refOGs (events)
OrthoLoger	164	61	58	59	20	18	12(50)
OrthoFinder	147	58	57	64	17	26	15(41)
SonicParanoid	163	62	63	62	19	23	11(53)
OrthoMCL	207	56	62	51	9	16	18(54)
OMA	325	56	65	27	4	0	16(62)

Besides the approaches to predict orthologs *ab-initio* in a set of genomes, one may want to map genes from a newly sequenced genome to pre-computed OGs. This provides a way to link to ortholog annotations in a database release and avoid skewing ortholog predictions for lower quality inputs, e.g. incomplete gene sampling from transcriptomes. OrthoLoger provides such a possibility. OrthoLoger is freely available from https://orthologer.ezlab.org.

## WEB INTERFACE

The web interface allows the OrthoDB database to be queried (Figure 1). By default, the search field keywords are used to retrieve the most relevant orthologous groups (OGs) containing these keywords anywhere in the corresponding gene and OG annotations. The search algorithm delivers OGs matching all keywords, after applying the search logic operators (see below). For both single keywords and phrases users can take advantage of a Google-like autocomplete lookup, self-activating after the first three characters of each word entered (Figure 1A). The autocomplete matches the characters case-sensitively anywhere in the word. This allows users to pin-point composite words, in addition to conventional left-anchored matches. For example, the search will return suggestions for various transferases [aminotransferase, methyltransferase, etc…] given ‘transferase’ as a prompt. The query can be more complex and supports logical operations to combine multiple keywords; for example ‘-’ or ‘!’ are interpreted as logical NOT that enables queries like [kinase !tyrosine]. To match a complete phrase one should use double quotation marks, e.g. [“Cytochrome P450”], as well as for querying EC numbers, e.g. [“3.1.1.-”]. Using the ‘Advanced’ panel one can filter the results for organismal taxonomy and/or the level of orthology by selecting the appropriate nodes on the species tree, and/or the member gene phyloprofile, e.g. present in >90% of the species (Figure 1A). The search algorithm matches OGs containing genes in ‘at least’ the organisms selected on the tree, usually with many others. For even more precision, it is possible to negate a certain clade in the above-mentioned taxonomic selection by an additional taxonomic node name in the text search widget, e.g. text search pattern ‘kinase !Metazoa’ with Eukaryota level selected delivers very specific kinases not present in Metazoa and similar organisms.

A specific ‘NCBI ID’ dropdown list was made for searching NCBI gene identifiers (aka gid), as many of them are just simple digits, like 1 or 9, hence would end up with spurious results if sought in a textual context among the entire body of annotation.

To enable users to save complex filtering setups for repetitive queries there is a link ‘Bookmark OrthoDB’ at the top-right corner that one can bookmark or drag & drop to the browser toolbar (Figure 1B). This link is actually a snippet of Javascript code, called bookmarklet, that allows for easy OrthoDB queries with the saved filter settings. Additionally, one can just highlight a keyword somewhere on a web page and click on the saved bookmarklet to search OrthoDB for the highlighted keyword.

To unambiguously navigate to a specific gene by its various identifiers one can select ‘get Gene’ in the dropdown in front of the search input (Figure 1C). The search then returns the best matching gene, expecting a pinpointing pattern, usually a gene identifier, either OrthoDB or an external one, e.g. Uniprot accession number P12345. Despite the enormous growth of covered sequence data OrthoDB still supports queries by a protein sequence. One can select ‘Sequence’ in the dropdown in front of the search input to effectively look for the best match using an amended Rapsearch aligner (40). This and the above-mentioned search modes return a gene-centric view, showing the organism of origin, collated gene annotations, a link to OGs containing this gene, and the list of pair-wise orthologs in example species, along with the genes’ annotations. In this release we added a search input to allow users to select an organism of interest for pair-wise orthologs (Figure 1D).

Orthology is used for many different goals. Arguably, the most common one is to get a hint about a particular gene's function. Navigating to a gene in OrthoDB by an identifier or by a sequence similarity search will reveal a consensus functional annotation of orthologs of this gene, bridging the experimental knowledge gained in model species and collated in OrthoDB to the other species. For example, one may wonder what is the importance of the PHUM213810 gene from the human body louse genome sequenced in 2010 beyond being a putative odorant receptor as inferred from a 7tm_6 Pfam signature. Querying OrthoDB for this identifier will reveal that it is a 1:1 ortholog of a well-studied fruit fly odorant receptor co-receptor (Orco) gene. The OrthoDB-computed evolutionary annotations of duplicability and universality could provide information about selection acting on these genes. In the case of Orco, it is found in a single copy in the vast majority of insects, hinting towards its essentiality (the appearance of multiple shorter genes in a few genomes suggests technical artifacts of fragmented gene predictions). One can further study the evolution of this gene family by retrieving protein sequences via the ‘Download Fasta’ link for this orthologous group and possibly homologous ones listed in the ‘Sibling Groups’ section, making a multiple sequence alignment with these protein sequences, and then building and exploring the gene tree. Instead of starting with a BLAST search seeking to gain functional or evolutionary insights into a protein of interest it may often be optimal to start by querying orthology databases. Conversely, querying OrthoDB for particular molecular function keywords may illuminate an evolutionary perspective on the underpinning genes. To make a species tree of a taxon one may want to extract sequences of single-copy orthologs. This can easily be done by going to the ‘Advanced’ panel of the OrthoDB web interface (Figure 1A), selecting the taxon of interest on the tree, e.g. Alveolata, selecting ‘present in >90% species’ and ‘single-copy in >90% species’ in the ‘Phyloprofile’ section, and retrieving the sequences for 252 Alveolata orthologous groups via ‘Download Fasta’.

## CONCLUSIONS AND PERSPECTIVES

This update of OrthoDB coverage to over 100 million genes from 18 000 prokaryotes and almost 2000 eukaryotes is a significant push forward of our previous record coverage. Moreover, sampling the genomic diversity for such complete orthology analysis paves the way for mapping of additional genomes at a fraction of the effort. The OrthoLoger software has proven its scalability and state-of-the-art accuracy, and its mapping mode provides users with an easy way to put additional genomes into the context of OrthoDB annotations. The demand for orthology will continue to increase with the growth of genomics and OrthoDB is striving to support it.

## DATA AVAILABILITY

The OrthoDB resource is public, including both data and data processing software. The optional registration allows authenticated users to upload their own proteomic data, for example from freshly sequenced genomes, for performing online BUSCO analysis and for mapping to the current OrthoDB data. This enables the user to map existing functional annotations to the new genes, as well as to generate user-tailored comparative charts depicting the total gene count, the fraction of common genes, the fraction of the most conserved single-copy genes, etc.

As for previous versions of OrthoDB we provide data files for bulk download, one file per level of orthology; as well as the underlying amino acid gene translations. To retrieve substantial subsets of data from OrthoDB or to access it programmatically we provide a REST API, documented at https://www.orthodb.org/orthodb_userguide.html#api, that returns data in *JSON*, *FASTA* or *TAB* formats. All data are distributed under the Creative Commons Attribution 3.0 License from https://www.orthodb.org/.

The RDF SPARQL interface uses URIs of UniProt proteins and Ensembl genes, to be compatible with both UniProt and Ensembl SPARQL endpoints, thus providing the possibility for very elaborate queries. Users can start exploring SPARQL code from a number of real-life biological examples (https://sparql.orthodb.org/) allowing retrieval of the genes along with a number of clickable links to Ensembl Genomes, NCBI, Interpro and GO resources. Users can also navigate to OrthoDB records by following links from FlyBase’s ‘Orthologs’ section, UniProt’s ‘Phylogenomic databases’ section, or NCBI’s ‘Additional links/ Gene LinkOut’ section.
